# Analysis of factors influencing urban residents’ environmental protection behavior

**DOI:** 10.3389/fpsyg.2025.1703070

**Published:** 2025-11-14

**Authors:** Yu He, Jintu Gu

**Affiliations:** School of Public Administration, Hohai University, Nanjing, China

**Keywords:** urban residents, environmental protection, influencing behavioral factors, combination model, DEMATEL-ISM-MICMAC

## Abstract

Against the backdrop of the “dual carbon” target becoming a national strategy, the environmentally friendly behavior of urban residents has become a key pivot to leverage ecological governance. However, in reality, although most urban residents identify with environmental protection concepts, they are constrained by multiple obstacles such as value conflicts, social norm pressures, economic cost considerations, and institutional contexts in daily practices such as garbage classification and low-carbon travel, forming deep bottlenecks that restrict the effectiveness of environmental governance. This study integrates the DEMATEL-ISM-MICMAC method to construct a three in one analytical framework of “driving mechanism transmission path governance strategy,” revealing the cascading impact chain from fundamental commitment to surface behavior, providing theoretical breakthroughs and practical paths for breaking the cognitive behavioral gap and achieving precise policy supply. The research results indicate that actual commitment (E8) serves as the fundamental driving force, with ultra-high driving force and extremely low dependence as the only independent factors, confirming that value internalization is the core engine of long-term environmental behavior; Environmental responsibility (E15) and civic behavior (E4) form a key hub node, with high centrality and strong interactivity linking the “cognition responsibility action” transformation chain; The surface level target ecological management (E1) exhibits significant passivity and is directly influenced by nine mid-level factors, highlighting the deep dependence of behavior implementation on systemic support. The dual low values of environmental emotion (E6) and verbal commitment (E7) expose their marginal position in the system, and pure emotional mobilization is difficult to activate the main behavioral chain. The four level transmission mechanism of E8 in the fundamental layer, E4/E15 in the middle layer, and E1/E16 in the surface layer further verifies that the improvement of environmental behavior efficiency needs to follow the progressive logic of “value foundation hub transmission terminal empowerment.” This study validates its practical effectiveness in promoting the transformation of residents from “cognitive identity” to “conscious action”, providing an operable and verifiable micro decision-making paradigm for the global urban carbon neutrality process, and promoting the paradigm shift of environmental governance research from factor identification to mechanism analysis and path optimization.

## 1 Introduction

In recent decades, China’s economy has achieved remarkable achievements that have attracted worldwide attention, but it has also caused a certain degree of damage to the ecological environment, sparking widespread discussions among countries around the world. At the 75th session of the United Nations General Assembly in 2020, the Chinese government pledged to “adopt more effective policies and measures to control carbon dioxide emissions, and achieve the goals of ‘carbon peak’ and ‘carbon neutrality’ by 2030 and 2060, respectively.” This shows that solving the environmental problems brought about by rapid economic development has become one of the important tasks of the Chinese government in the future period (Jingcheng and Donglin, 2024). In this context, urban residents are no longer just passive bearers of environmental pressure, but their daily consumption decisions and environmental behavior habits, such as garbage classification, resource conservation, and low-carbon travel^1^. Waiting has become a key pivot to leverage the urban environmental governance system, profoundly shaping the urban ecosystem^2^ Health and resilience. However, there is still a significant gap between the theoretical understanding of “environmental awareness” among urban residents and the actual adoption of environmentally friendly behaviors. Although most residents agree with the importance of environmental protection in terms of ideology, the frequency, depth, and sustainability of their pro environmental behavior in complex daily situations are deeply constrained and influenced by multidimensional and multi-level complex factors, involving individual values, social normative pressure, economic cost considerations, situational convenience conditions, structural institutional arrangements, and many other aspects (Xianshi, 2025). This profound cognitive behavioral difference has become the core bottleneck restricting the improvement of urban environmental governance efficiency. Therefore, it is necessary to conduct in-depth investigations into the environmental protection behavior of urban residents in China. Based on this, this study takes Jiangsu Province as the research object, analyzes the various factors that affect the environmental protection behavior of urban residents, and then formulates practical and feasible strategies to stimulate the enthusiasm of urban residents to implement environmental protection behavior, providing assistance for the improvement of the level of urban environmental protection work.

Kaisera, based on theories of environmental psychology and social psychology, used survey questionnaires and observation methods to analyze residents’ behavior towards the environment. She believed that human behavior to some extent represents human attitudes, and evaluated people’s attitudes towards the environment by analyzing their behavior towards the environment. Based on environmental behavior and applied psychology (Du Nann Winter and Koger, 2004), Steg adopts a combination of qualitative and quantitative methods to study that human environmental behavior has a decisive impact on environmental quality (Gardner and Stern, 2002). Firstly, assess the behavior of change; secondly, explore the specific factors that trigger these behaviors; again, develop specific strategies to influence environmental behavior; finally, evaluate whether the strategy has played its due role. Based on the theories of environmental sociology and cross-cultural psychology, Wang pointed out in his research that urban–rural differences, value orientations, and environmental awareness are the main factors affecting environmental protection behavior. The above research confirms that environmental awareness, economic income, education, emotions, and other factors have a significant impact on environmental protection behavior, but the analysis has not been conducted from an institutional perspective, and the conclusions drawn have certain limitations (Vlek and Steg, 2007). At present, although relevant research has extensively explored the factors influencing urban residents’ environmental protection behavior from multiple perspectives such as social structure, demographic statistics, and psychology, these analyses often present a fragmented state and have not been integrated into a unified analytical framework. This theoretical deficiency not only highlights the research gap in the current field, but also hinders the improvement of environmental governance efficiency. The DEMATEL-ISM-MICMAC method breaks through the boundary of a single model through the organic integration of decision-making laboratory analysis, interpretation structure model and cross influence matrix, and constructs an analytical framework of “driving mechanism transmission path governance strategy.” This method accurately identifies key driving factors, deeply analyzes the complete transmission path from individual cognition, social norms to behavioral decision-making, sorts out the internal laws and key transmission nodes of how different levels and attribute factors interact and ultimately affect behavioral performance, and reveals the dynamic mechanism of behavior. Finally, based on a deep understanding of the transmission path and its key links, differentiated comprehensive governance strategies with strong targeting and hierarchical matching are proposed, providing micro decision support and more operational precise policy supply paths for urban environmental governance, and promoting the refined transformation of relevant governance paradigms.

## 2 Identification of influencing factors, data sources, and research methods

### 2.1 Identification of influencing factors

There are two main ways to identify influencing factors in existing research: one is to directly identify influencing factors through expert questionnaires; the second is to identify the influencing factors through existing literature. To avoid subjective bias, this study identified the influencing factors through existing domestic and foreign literature (Environmental Research, 2020). And based on the extracted influencing factor attributes, it is divided into five categories: environmental behavior, environmental awareness, environmental knowledge, external factors, and personality variables. The specific classification categories and corresponding influencing factors can be found in Table 1.

**Table 1:** Factors influencing urban residents’ environmental protection behavior.

Category	Theoretical framework	Influencing factors	Meaning and reference sources
Environmental behavior	Theory of planned behavior (Mohammad et al., 2022)	Ecological management (E1)	Urban residents, in order to protect the ecological environment they rely on for survival, improve their economic development level without affecting the ecological environment, achieve sustainable development goals, implement green lifestyles, save resources, and avoid wasteful behavior (Hines et al., 1987).
		Consumer behavior (E2)	Urban residents, with the aim of reducing pollution and protecting the ecological environment, engage in activities such as screening, purchasing, and experiencing various consumer goods and services to meet their daily needs. The consumption behavior of urban residents based on environmental protection plays an important role in improving the quality of urban environment and ensuring harmonious coexistence between urban residents and the environment without compromising their quality of life (Hsu and Roth, 1998).
		Persuasive behavior (E3)	Does urban residents intervene in behaviors that damage the environment and persuade them to do so in the process of urban life (Kaisera et al., 2007).
		Citizen Behavior (E4)	Urban residents’ sense of social responsibility towards environmental protection, participation in social activities, etc. (Steg and Vlek, 2009).
Environmental awareness	Value belief norm theory (Qiu, 2024; Jia et al., 2018)	Individual environmental cognition (E5)	It is the knowledge about environmental protection that one possesses (Feng and Reisner, 2011).
		Environmental emotions (E6)	An individual’s lasting emotional experience and physiological evaluation of the natural environment and their own environmental behavior, which includes positive emotions (such as love for nature, pride in environmental behavior) and negative emotions (such as guilt over environmental damage, concern about pollution). Current research generally recognizes that environmental emotions are a key factor driving pro environmental behavior, and their impact often goes beyond cognitive factors. Scholars have constructed multidimensional measurement models that include feelings of worry, passion, guilt, and found that they play a central role in the dilemma of “knowing is easier than doing” by stimulating motivation. For example, close range environmental pollution can effectively stimulate emotions and promote behavioral change (Picherta and Katsikopoulos, 2008).
		Verbal commitment (E7)	That is, the willingness to take action (Davis et al., 2009).
		Actual commitment (E8)	Individual participation in environmental protection behaviors (Sia et al., 1985/1986).
Environmental knowledge	Knowledge attitude behavior model (Wang and Zhang, 2021; Gu et al., 2023)	Natural environment knowledge (E9)	Basic cognitive information about the basic composition, operational laws, interrelationships, and inherent value of the Earth’s natural ecosystems, and understanding their structure and function (Smith-Sebasto and D’ Acosta, 1995).
		Environmental knowledge (E10)	Understand the specific information on the causes, scale, consequences, and urgency of various ecological environment deterioration phenomena caused by human activities or natural changes, and understand the essence of the problem (Stem, 2000).
		Environmental action knowledge (E11)	Know the relevant knowledge and skill information of specific and effective behavioral strategies, methods, and solutions that individuals or groups can adopt to alleviate environmental problems and protect the ecology, and master participation methods (Ruth Rogana et al., 2005).
External factors	Social cognitive theory (Zhao et al., 2021) and institutional theory (Li, 2023)	Difficulty of behavior (E12)	When individuals implement environmentally friendly behaviors, objective situational conditions such as perceived or actual convenience, the amount of effort required, and the convenience of obtaining relevant resources or information are considered (Fletcher et al., 2009).
		Social regulations (E13)	The institutional environment in which environmental protection laws, regulations, policies formulated by the government and informal norms established by communities or organizations (such as conventions and customs) guide or constrain behavior through reward and punishment mechanisms (André Hansla and Asgeir Juliusson, 2008).
		Economic conditions (E14)	The level of economic resources possessed by an individual or family directly affects their ability and willingness to bear the cost of environmentally friendly products or services, such as the premium for energy-saving appliances and public transportation expenses (Sia et al., 1985/1986).
Personality variables	Standardized activation model (Xie and Xu, 2024; Zhao and Tian, 2023; Liu et al., 2022)	Environmental responsibility (E15)	Sense of responsibility is the primary antecedent variable that influences environmental behavior (Smith-Sebasto and D'Acosta, 1995).
		Sense of environmental control (E16)	People with a sense of internal control are more inclined to adopt environmental behaviors (Maloney and Ward, 1973).

### 2.2 Data sources

Design a survey questionnaire based on the 16 influencing factors listed in the table above, which includes 240 questions about the relative impact of pairwise factors, such as “How much do you think ecological management affects consumer behavior,” “How much do you think ecological management affects persuasive behavior,” “How much do you think ecological management affects citizen behavior,” “How much do you think ecological management affects individual environmental cognition,” and so on. Choose the Likert five level scale to quantitatively evaluate the degree of influence between different influencing factors. The degree of impact is divided into five levels, namely “no impact (0 points),” “low impact (1 point),” “moderate impact (2 points),” “high impact (3 points),” and “extremely high impact (4 points).” Specifically, a score of 0 represents’ no impact ‘, meaning there is no identifiable causal relationship or influence path between the two factors. 1 point corresponds to ‘low impact’, indicating that factor A has a slight impact on factor B, but this impact is relatively weak and not a critical driving factor. A score of 2 represents’ moderate impact ‘, indicating that one factor has a clear and significant moderate impact on another factor, and is one of the forces in the system. A score of 3 represents’ higher impact ‘, indicating that factor A has a significant driving effect on factor B and is an important prerequisite for its changes. The highest 4 points correspond to ‘extremely high impact’, used to identify those driving relationships that are decisive and strong, where factor A plays a crucial core role in the changes of factor B.

This survey adopts an online survey method, sending scoring questionnaires to 20 experts (the selection criteria for experts are shown in Table 2), and providing detailed explanations of each influencing factor in the email, so that they can score the degree of influence of each factor. Finally, the collected questionnaires are summarized and organized to obtain various data required for the research.

**Table 2:** Expert selection criteria.

Standard dimension	Specific standards
Minimum working years	5 years or more
Scope of professional field	Environmental science, environmental engineering, public management, sociology, behavioral psychology
Occupation	Scholars from universities and research institutions, officials from government environmental protection departments, and leaders of environmental social organizations
Educational status	Master’s degree or above

Experts selected under this standard have profound academic backgrounds and rich practical experience, which enables the scoring process to comprehensively consider theoretical frontiers, policy feasibility, and social acceptance, effectively avoiding the limitations of a single perspective. It is precisely this professional authority supported by high education, long years of experience, and a wide range of fields that provides high-quality data input for the complex calculations of the DEMATEL-ISM-MICMAC combination model in the future, ensuring the scientific and robust nature of the entire research analysis process and conclusions. At the same time, in order to ensure the reliability of the research results, SPSSAU software was used to conduct a reliability test on the opinions of experts, and the result was 0.8863, which is greater than 0.80, indicating that the questionnaire has good internal consistency and the reliability of the research results is high. To eliminate individual differences, the scores are averaged and rounded to obtain the direct impact matrix.

### 2.3 Research methods

This study applies the DEMATEL-ISM-MICMAC method to the study of factors influencing urban residents’ environmental protection behavior, aiming to clarify the logical relationship between various influencing factors.

#### 2.3.1 DEMATEL method analysis steps

1. Establish an initial direct impact matrix Z based on the survey results, sum up all scoring data, and calculate the average value of each unit. Use Python 3.11 program to solve the matrix and obtain the initial direct impact matrix Z, Z = [Aij], where Aij represents the degree of influence of factor i on factor j (Fang et al., 2023).
2. Calculate the standardized direct impact matrix X and the comprehensive impact matrix T separately. First, use the maximum normalization method to standardize the initial direct impact matrix Z. Specifically, divide the values in the initial direct impact matrix by the maximum sum of each row, and then complete the standardization process (Xu et al., 2023).

Step 1: Calculate the sum of each row of the initial matrix Z. Let the initial direct impact matrix Z be an n × n matrix, where element Z_ {ij} represents the degree of direct impact of factor i on factor j. Calculate the sum of each row of elements:


$$R_{i}=\sum_{j=1}^{n}Z_{\mathrm{ij}}(i=1,2\ldots n)$$

Step 2: Determine the normalization factor *λ*. Take the maximum value among all rows and as the normalization factor:


$$\lambda =\max_{i}(R_{i})$$

Step 3: Calculate the standardized matrix X. Divide each element in Z by λ to obtain the standardized direct impact matrix X.


$$X_{\mathrm{ij}}=\frac{Z_{\mathrm{ij}}}{\lambda }$$

Calculate the comprehensive impact matrix T:


T=T(1−X)−1.

In the formula, I is the identity matrix, and (I-X) -1 is the inverse matrix of (I-X).

3 Determine centrality and causality

Based on the comprehensive influence matrix T, the influence degree, affected degree, centrality, and causal degree of each influencing factor can be obtained. Among them, centrality represents the importance and impact of the factor, and is a positive correlation; Reason degree represents the degree of mutual influence between two factors. Generally speaking, if the value of reason degree is above 0, it indicates that the factor can affect other factors. These factors are called cause factors, and the larger the value, the higher the degree of influence. Conversely, if the value of reason degree is below 0, it indicates that the factor is easily influenced by other factors. These factors are called result factors, and the magnitude of the value is negatively correlated with the degree of influence (Feng et al., 2023).

#### 2.3.2 Analysis steps of ISM method

1 Establish an overall impact matrix H

The comprehensive impact matrix constructed using DEMATEL ignores the impact of factors on itself, so it is necessary to introduce an identity matrix, represented as I, and add the identity matrix to the comprehensive impact matrix to construct the overall impact matrix, represented as:


H=I+T

2 Generate reachable matrix M

The so-called reachable matrix, in simple terms, refers to whether there is a connection path between two factors. If there is, it is represented as 1, otherwise it is represented as 0 (Qingqing and Xiaofang, 2024). This article constructs a reachable matrix based on the comprehensive impact matrix T. In order to avoid the system being too complex, it is necessary to select appropriate thresholds and eliminate some factors with lower impact. T has a decisive impact on the system structure. If the threshold is too large, the system structure is too simple to accurately determine the interrelationships between different factors. Conversely, if the threshold is too small, the system structure will be too complex and difficult to use. Therefore, the value of T is very important. In addition, the threshold size has a significant impact on the hierarchical division of the explanatory structural model, so it is necessary to consider comprehensively when setting the threshold.

Generally speaking, there are three main methods used when setting thresholds. The first method is the empirical value method (Li et al., 2025). Simply put, it is based on the past experience of experts and scholars to select an appropriate threshold. For systems with relatively few influencing factors, the threshold can be set to “0” because there is no need to simplify the system structure. In terms of this study, due to the numerous factors that affect the environmental protection behavior of urban residents and the complex relationships between different factors, coupled with the limited number of experts and scholars who have chosen this method for research, it is difficult to obtain appropriate threshold values through this method.

The second method is the metrological inspection method. This method has relatively high requirements for preliminary data investigation. If the obtained data is not accurate and complete enough, appropriate thresholds cannot be obtained (Zhou et al., 2025).

The third method is the average method. Recently, more and more scholars have begun to try using the average value and standard deviation of the comprehensive influence matrix to select thresholds. Generally speaking, models with more influencing factors choose this method to select thresholds, which is more appropriate and has lower requirements for previous investigations. Therefore, after comprehensive consideration, this study finally adopted this method to set thresholds (Jia, 2025).

3 Divide the hierarchy of influencing factors. Firstly, using the reachability matrix, the influencing factors are divided into different levels, obtaining the reachability set (R), the antecedent set (Q), and the intersection (C). Then, based on the hierarchical division results, each influencing factor is decomposed into different levels. Specifically, according to the decomposition rule R ∩ Q = C = R, the eligible influencing factors are classified into the first level. After that, the influencing factors selected in the first screening are removed and screened again, and a hierarchical decomposition table is established.
4 Establish an explanatory structural model. Using Visio2021 to construct an explanatory structural model, and based on the hierarchical division of influencing factors, identifying influencing factors, indirect factors, and fundamental factors.

#### 2.3.3 Analysis steps of MICMAC method

**Figure 1:**
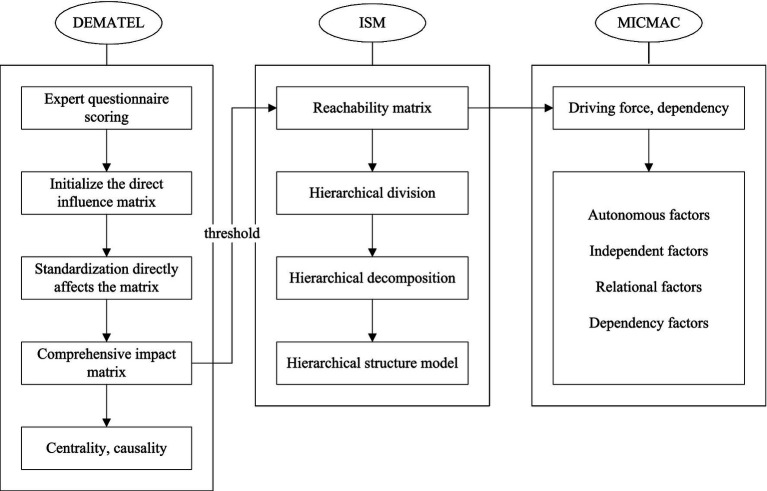
Analysis steps of DEMATEL-ISM-MICMAC method.

The MICMAC method is a widely used method for factor classification, which can accurately determine the role of each factor in system operation and the relationships between different factors. Sum up each row and column of the reachable matrix to obtain the values of driving force and dependency for each factor. Use Excel to draw a quadrant chart with the horizontal and vertical axes representing driving force and dependency, and set the mean of the driving force and dependency chart as the boundary (Wang et al., 2020). The quadrant diagram divides influencing factors into four categories: autonomous factors, dependent factors, correlated factors, and independent factors. As for autonomous factors, their driving force and dependence are relatively low, located in quadrant I; In terms of dependency factors, it has a high degree of dependence but a low driving force, located in the fourth quadrant; In terms of related factors, their dependence and driving force are relatively high, located in the third quadrant; In terms of independent factors, their dependence is low, while their driving force is high, located in the second quadrant. Figure 1 provides a more intuitive demonstration of the steps for using the DEMATEL-ISM-MICMAC method.

## 3 Results analysis

### 3.1 Analysis of expert investigation situation

The distribution of questionnaires to experts is shown in Table 3. From the table, it can be seen that the average age of the expert team is 51.7 years old, presenting a team structure centered around experienced senior experts. Specifically, the age range of team members is from 38 to 64 years old, forming a good hierarchical distribution. Among them, experts under 45 years old account for 25%, experts between 45 and 55 years old account for 40%, and senior experts over 55 years old account for 35%. This age structure, which combines the elderly, middle-aged, and young, not only ensures the maturity and reliability of professional judgment, but also stems from the profound knowledge and rich practical experience accumulated by senior experts in the field of environment over the years; It has injected new vitality and cutting-edge perspectives into the team, and young and middle-aged experts often maintain a higher sensitivity to emerging technologies and methods. In terms of work experience, the average tenure of experts is 25.6 years, and the vast majority of members have over 15 years of industry experience, which lays a solid foundation for ensuring the accuracy of research data scoring and judgment.

**Table 3:** Basic information of surveyed experts.

Serial number	Name	Age	Gender	Occupation category	Professional field	Highest degree	working hours
1	Zhang XX	48	Male	University faculty	Water pollution control	Ph.D.	22
2	Wang XX	61	Male	Researcher at a research institution	Atmospheric Environmental Chemistry	Ph.D.	35
3	Li XX	45	Female	Senior Engineer of Environmental Protection Enterprise	Resource utilization of solid waste	Master’s degree	18
4	Liu XX	55	Male	Experts from government environmental departments	Environmental Planning and Management	Master’s degree	28
5	Chen XX	50	Male	university faculty	Soil ecological restoration	Ph.D.	23
6	Zhao XX	42	Female	Senior Engineer of Environmental Protection Enterprise	environmental monitoring	Master’s degree	17
7	Huang XX	57	Male	Researcher at a research institution	Prevention and control of heavy metal pollution	Ph.D.	30
8	Zhou XX	63	Male	university faculty	Air pollution prevention and control	Ph.D.	38
9	Wu XX	38	Female	Experts from government environmental departments	Environmental Impact Assessment	Ph.D.	12
10	Sun XX	49	Male	Senior Engineer of Environmental Protection Enterprise	sewage treatment	Master’s degree	22
11	Money XX	58	Male	university faculty	New energy technology	Ph.D.	32
12	Zheng XX	43	Female	Senior Engineer of Environmental Protection Enterprise	cleaner production	Master’s degree	18
13	Ma XX	62	Male	Researcher at a research institution	ecological protection	Ph.D.	36
14	GaoXX	52	Male	Experts from government environmental departments	environmental policy	Master’s degree	25
15	Lin XX	56	Female	university faculty	Environmental Health	Ph.D.	29
16	He XX	47	Male	Senior Engineer of Environmental Protection Enterprise	Environmental materials	Ph.D.	19
17	Zhu XX	64	Male	Researcher at a research institution	climate change	Ph.D.	39
18	Qin XX	50	Female	Senior Engineer of Environmental Protection Enterprise	Environmental Engineering	Master’s degree	24
19	Song XX	58	Male	University faculty	Environmental Law	Ph.D.	31
20	Dong XX	46	Male	Experts from government environmental departments	Environmental Economy	Master’s degree	18

### 3.2 Analysis of DEMATEL model results

1 Establish the initial direct impact matrix Z

Calculate the average of 20 scoring data points as the initial direct impact matrix for urban residents’ environmental protection behavior factors, Z = [Aij]n*n, among them, i represents the i-th row factor, j represents the j-th column factor, Aij represents the degree of influence of factor i on factor j, and initializing the direct impact matrix can reflect the direct impact relationship between various factors. As shown in Table 4.

2 Calculate the standardized direct impact matrix X and the comprehensive impact matrix T

**Table 4:** Initial direct impact matrix Z.

	E1	E2	E3	E4	E5	E6	E7	E8	E9	E10	E11	E12	E13	E14	E15	E16
E1	0.00	1.75	1.90	2.10	2.05	2.05	1.95	2.05	2.05	2.00	2.25	2.05	2.10	2.00	2.15	2.20
E2	2.15	0.00	2.30	2.25	2.05	2.05	2.05	1.95	2.00	2.00	2.05	2.00	2.15	2.40	2.25	2.15
E3	2.05	2.25	0.00	2.10	2.05	2.05	2.05	2.05	2.05	2.05	2.05	2.05	2.15	2.10	2.05	2.15
E4	2.40	2.20	2.20	0.00	2.05	1.95	2.05	2.05	2.20	2.25	2.25	2.20	2.00	2.20	2.25	2.35
E5	2.45	1.95	2.10	2.45	0.00	2.05	1.95	2.05	2.10	2.05	2.10	2.25	2.25	2.00	2.15	2.05
E6	2.25	2.20	2.05	1.90	2.05	0.00	2.05	1.95	2.00	2.20	1.90	2.05	2.05	2.10	2.25	2.25
E7	1.95	1.85	2.00	1.80	2.05	2.05	0.00	2.05	2.15	2.10	2.10	2.10	2.10	2.15	2.05	1.85
E8	2.25	2.25	2.40	2.45	1.95	2.05	2.05	0.00	2.05	2.10	2.10	2.30	2.05	2.20	2.15	2.10
E9	1.90	2.20	1.80	1.95	2.05	1.95	2.05	2.05	0.00	2.05	2.10	2.10	2.20	2.05	2.15	2.25
E10	2.30	1.95	2.35	2.15	2.10	2.05	1.95	2.05	2.00	0.00	2.15	2.05	2.10	2.00	2.25	2.20
E11	2.30	2.10	2.05	2.00	2.05	2.05	2.05	1.95	2.10	1.95	0.00	2.05	2.20	2.00	2.20	2.10
E12	1.80	1.80	1.95	1.90	1.95	2.05	2.05	2.05	2.00	2.05	2.15	0.00	1.90	2.05	2.10	2.40
E13	2.40	2.35	2.10	2.25	2.05	1.95	2.05	2.05	2.10	2.25	2.20	2.20	0.00	2.25	2.20	2.05
E14	1.90	2.10	2.20	2.05	2.05	2.05	1.95	2.05	1.95	2.00	2.05	2.00	2.00	0.00	1.95	1.95
E15	2.80	2.20	2.30	2.50	2.05	2.05	2.05	1.95	2.15	2.30	2.05	2.25	2.15	2.15	0.00	2.50
E16	1.90	2.00	1.75	1.95	1.95	2.05	2.05	2.05	2.05	2.00	2.05	2.10	2.05	2.10	2.35	0.00

Using maximum normalization to standardize the initial direct impact matrix, the standardized direct impact matrix X is obtained. Specifically, the values contained in the matrix are divided by the sum of each row to complete the standardization of the initial direct impact matrix Z. That is to say, divide the sum of each number in the first row of Table 4 by the sum of the sum, and obtain the matrix in Table 5. At the same time, it is necessary to calculate the comprehensive impact matrix based on the standardized direct impact matrix, in order to accurately determine the indirect relationship between various influencing factors. Tables 5, 6 provide detailed data for these two matrices.

3 Determine centrality and causality

**Table 5:** Standardization direct impact matrix X.

	E1	E2	E3	E4	E5	E6	E7	E8	E9	E10	E11	E12	E13	E14	E15	E16
E1	0.000	0.052	0.057	0.063	0.061	0.061	0.058	0.061	0.061	0.060	0.067	0.061	0.063	0.060	0.064	0.066
E2	0.064	0.000	0.069	0.067	0.061	0.061	0.061	0.058	0.060	0.060	0.061	0.060	0.064	0.072	0.067	0.064
E3	0.061	0.067	0.000	0.063	0.061	0.061	0.061	0.061	0.061	0.061	0.061	0.061	0.064	0.063	0.061	0.064
E4	0.072	0.066	0.066	0.000	0.061	0.058	0.061	0.061	0.066	0.067	0.067	0.066	0.060	0.066	0.067	0.070
E5	0.073	0.058	0.063	0.073	0.000	0.061	0.058	0.061	0.063	0.061	0.063	0.067	0.067	0.060	0.064	0.061
E6	0.067	0.066	0.061	0.057	0.061	0.000	0.061	0.058	0.060	0.066	0.057	0.061	0.061	0.063	0.067	0.067
E7	0.058	0.055	0.060	0.054	0.061	0.061	0.000	0.061	0.064	0.063	0.063	0.063	0.063	0.064	0.061	0.055
E8	0.067	0.067	0.072	0.073	0.058	0.061	0.061	0.000	0.061	0.063	0.063	0.069	0.061	0.066	0.064	0.063
E9	0.057	0.066	0.054	0.058	0.061	0.058	0.061	0.061	0.000	0.061	0.063	0.063	0.066	0.061	0.064	0.067
E10	0.069	0.058	0.070	0.064	0.063	0.061	0.058	0.061	0.060	0.000	0.064	0.061	0.063	0.060	0.067	0.066
E11	0.069	0.063	0.061	0.060	0.061	0.061	0.061	0.058	0.063	0.058	0.000	0.061	0.066	0.060	0.066	0.063
E12	0.054	0.054	0.058	0.057	0.058	0.061	0.061	0.061	0.060	0.061	0.064	0.000	0.057	0.061	0.063	0.072
E13	0.072	0.070	0.063	0.067	0.061	0.058	0.061	0.061	0.063	0.067	0.066	0.066	0.000	0.067	0.066	0.061
E14	0.057	0.063	0.066	0.061	0.061	0.061	0.058	0.061	0.058	0.060	0.061	0.060	0.060	0.000	0.058	0.058
E15	0.084	0.066	0.069	0.075	0.061	0.061	0.061	0.058	0.064	0.069	0.061	0.067	0.064	0.064	0.000	0.075
E16	0.057	0.060	0.052	0.058	0.058	0.061	0.061	0.061	0.061	0.060	0.061	0.063	0.061	0.063	0.070	0.000

**Table 6:** Comprehensive impact matrix T.

	E1	E2	E3	E4	E5	E6	E7	E8	E9	E10	E11	E12	E13	E14	E15	E16
E1	0.927	0.933	0.944	0.959	0.923	0.922	0.917	0.919	0.935	0.945	0.957	0.957	0.950	0.955	0.980	0.983
E2	1.020	0.914	0.986	0.995	0.954	0.953	0.950	0.947	0.965	0.976	0.983	0.987	0.983	0.998	1.014	1.014
E3	1.002	0.962	0.907	0.976	0.939	0.938	0.935	0.935	0.952	0.962	0.968	0.973	0.968	0.974	0.994	0.998
E4	1.049	0.997	1.005	0.954	0.975	0.971	0.970	0.970	0.991	1.004	1.010	1.014	1.000	1.014	1.037	1.041
E5	1.033	0.973	0.985	1.005	0.900	0.957	0.951	0.954	0.972	0.982	0.989	0.998	0.990	0.991	1.016	1.016
E6	1.007	0.960	0.965	0.971	0.939	0.880	0.935	0.932	0.950	0.966	0.964	0.973	0.965	0.974	0.999	1.001
E7	0.973	0.927	0.939	0.943	0.915	0.914	0.853	0.911	0.930	0.939	0.944	0.949	0.941	0.951	0.968	0.965
E8	1.041	0.994	1.007	1.018	0.968	0.970	0.967	0.909	0.984	0.996	1.002	1.013	0.998	1.010	1.030	1.031
E9	0.987	0.950	0.947	0.961	0.929	0.925	0.925	0.925	0.883	0.952	0.959	0.964	0.958	0.962	0.985	0.990
E10	1.020	0.965	0.984	0.989	0.951	0.949	0.943	0.946	0.961	0.916	0.982	0.984	0.977	0.983	1.011	1.011
E11	1.006	0.955	0.962	0.971	0.937	0.935	0.933	0.930	0.950	0.957	0.908	0.970	0.966	0.969	0.995	0.994
E12	0.965	0.921	0.933	0.941	0.908	0.910	0.907	0.907	0.921	0.933	0.941	0.886	0.932	0.944	0.965	0.975
E13	1.045	0.997	0.999	1.013	0.971	0.967	0.967	0.967	0.985	1.000	1.005	1.010	0.940	1.011	1.031	1.029
E14	0.970	0.931	0.941	0.947	0.913	0.911	0.906	0.909	0.922	0.934	0.941	0.944	0.936	0.888	0.963	0.965
E15	1.083	1.019	1.031	1.047	0.997	0.996	0.993	0.990	1.013	1.028	1.028	1.038	1.027	1.036	0.997	1.069
E16	0.974	0.933	0.934	0.949	0.914	0.916	0.913	0.913	0.929	0.938	0.945	0.951	0.942	0.951	0.978	0.914

**Figure 2:**
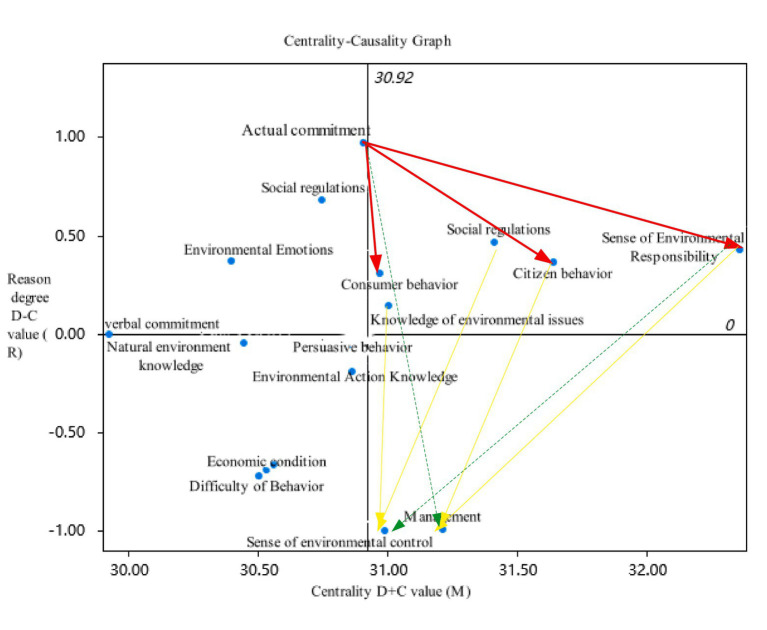
Centrality causality diagram. The red thick arrow represents the fundamental factor path; the yellow arrow represents the path of associated factors; the green dashed arrow represents the cross level path.

Calculate the sum of each row and column in the comprehensive impact matrix T, represented as ri and ci, respectively, representing the comprehensive impact of the factor on other factors and the comprehensive impact of other factors. The sum of the two is the centrality of the factor, and the magnitude of the centrality value is positively correlated with the impact and importance of the factor; the result of subtracting the two is the causal degree of the factor. If the value of the causal degree is above 0, it indicates that the factor has a prominent impact on other factors and is also known as the causal factor; On the contrary, if the value is below 0, it indicates that the factor will be influenced by other factors, also known as the result element. Table 7 details the centrality and causality values of each influencing factor, while Figure 2 intuitively shows the position of the centrality and causality of each influencing factor.

**Table 7:** Centrality and causality values.

	Impact degree ri	ranking	Affected degree ci	ranking	Centrality ri + ci	ranking	Reason degree ri ci	ranking
Ecological management (E1)	15.106	11	16.102	1	31.208	4	−0.995	15
Consumer behavior (E2)	15.639	5	15.331	10	30.969	7	0.308	7
Persuasive behavior (E3)	15.384	7	15.468	8	30.852	10	−0.085	11
Citizen behavior (E4)	16.000	2	15.637	4	31.638	2	0.363	6
Individual environmental cognition (E5)	15.712	4	15.034	12	30.746	11	0.678	2
Environmental emotions (E6)	15.381	8	15.012	13	30.392	15	0.369	5
Verbal commitment (E7)	14.962	13	14.964	15	29.926	16	−0.003	9
Actual commitment (E8)	15.938	3	14.965	14	30.903	8	0.973	1
Natural environment knowledge (E9)	15.200	10	15.244	11	30.443	14	−0.044	10
Environmental knowledge (E10)	15.572	6	15.428	9	31.001	5	0.144	8
Environmental action knowledge (E11)	15.337	9	15.526	6	30.863	9	−0.189	12
Difficulty of behavior (E12)	14.889	15	15.612	5	30.501	13	−0.723	14
Social regulations (E13)	15.938	3	15.472	7	31.411	3	0.466	3
Economic conditions (E14)	14.921	14	15.612	5	30.533	12	−0.691	13
Environmental responsibility (E15)	16.392	1	15.963	3	32.354	1	0.429	4
Sense of environmental control (E16)	14.994	12	15.995	2	30.989	6	−1.001	16

Firstly, analyze the centrality of each influencing factor, which is the result of adding the influence value and the influenced value. The centrality value reflects the importance level of the influencing factor, and the two are positively correlated. According to the centrality value ranking results, from highest to lowest, they are environmental responsibility (E15), civic behavior (E4), social regulations (E13), ecological management (E1), environmental problem knowledge (E10), environmental control (E16), consumption behavior (E2), actual commitment (E8), environmental action knowledge (E11), persuasive behavior (E3), individual environmental cognition (E5), economic conditions (E14), behavioral difficulty (E12), and nature. Environmental knowledge (E9), environmental emotions (E6), and verbal commitments (E7). The factors with high centrality values include environmental responsibility (E15), civic behavior (E4), social regulations (E13), ecological management (E1), environmental knowledge (E10), environmental control (E16), and consumer behavior (E2). These factors are indispensable in influencing urban residents’ environmental protection behavior and are closely related to other factors, making them important factors affecting residents’ environmental protection behavior.

Secondly, analyze the causal degree of each influencing factor, which is the result of subtracting the influential degree value from the influenced degree value. Divide the influencing factors into causal factors and causal factors with 0 as the dividing line. Factors above 0 are called causal factors, which have a relatively strong impact on other factors; Factors below 0 are called outcome factors, which are influenced by other factors. The ranking of reasons in descending order is as follows: actual commitment (E8), individual environmental cognition (E5), social regulations (E13), environmental responsibility (E15), environmental emotions (E6), civic behavior (E4), consumer behavior (E2), environmental knowledge (E10), verbal commitment (E7), natural environmental knowledge (E9), persuasive behavior (E3), environmental action knowledge (E11), economic conditions (E14), behavioral difficulty (E12), ecological management (E1), and environmental control (E16). There are a total of five causal factors, including environmental responsibility (E15), social regulations (E13), civic behavior (E4), consumer behavior (E2), and environmental knowledge (E10). These five factors have a prominent impact on other factors, so it is important to focus on these factors and develop corresponding strategies to improve the environmental protection level of urban residents. There are five outcome factors, namely natural environmental knowledge (E9), persuasive behavior (E3), environmental behavior knowledge (E11), economic conditions (E14), and behavioral difficulty (E12), which are greatly influenced by other factors. The reason degree value of environmental control sense (E16) is the smallest, and compared with other factors, the gap is also relatively large. This also means that this factor will be more prominently influenced by other factors. Developing corresponding strategies for this factor has a significant promoting effect on improving environmental protection effectiveness.

### 3.3 Analysis of ISM model results

1 Generate reachable matrix M

Based on the comprehensive influence matrix T and threshold, a reachable matrix M is constructed. The threshold is the sum of the mean and standard deviation of the comprehensive influence matrix, which are 0.9662 and 0.068, respectively. Therefore, based on the output model, the threshold is set to 0.97, and the calculation formula is: mean 0.9662 + standard deviation 0.068. The mean represents the average strength of all influence relationships in the comprehensive influence matrix, while the standard deviation quantifies the degree of dispersion of these influence relationships. The purpose of using the standard of “mean plus standard deviation” is to screen out those significant impact relationships that are higher than the average level, thereby effectively focusing on key factors, simplifying the system structure, and enhancing the model’s identification ability. The overall impact matrix H is the sum of the comprehensive impact matrix T and the identity matrix I. If the value is 1 or above, then it takes the value 1, otherwise it takes the value 0. This method can be used to calculate the reachable matrix M. Table 8 provides detailed results of the values.

2 Hierarchical division and decomposition of influencing factors

**Table 8:** Reachable matrix table M.

	E1	E2	E3	E4	E5	E6	E7	E8	E9	E10	E11	E12	E13	E14	E15	E16
E1	1	0	0	0	0	0	0	0	0	0	0	0	0	0	0	0
E2	1	1	1	1	1	0	0	0	1	1	1	1	1	1	1	1
E3	1	0	1	0	0	0	0	0	0	0	0	0	0	0	0	0
E4	1	1	1	1	1	0	0	0	1	1	1	1	1	1	1	1
E5	1	1	1	1	1	0	0	0	1	1	1	1	1	1	1	1
E6	1	0	0	0	0	1	0	0	0	0	0	0	0	0	0	1
E7	0	0	0	0	0	0	1	0	0	0	0	0	0	0	0	0
E8	1	1	1	1	1	0	0	1	1	1	1	1	1	1	1	1
E9	0	0	0	0	0	0	0	0	1	0	0	0	0	0	0	0
E10	1	1	1	1	1	0	0	0	1	1	1	1	1	1	1	1
E11	1	0	0	0	0	0	0	0	0	0	1	0	0	0	0	0
E12	0	0	0	0	0	0	0	0	0	0	0	1	0	0	0	0
E13	1	1	1	1	1	0	0	0	1	1	1	1	1	1	1	1
E14	0	0	0	0	0	0	0	0	0	0	0	0	0	1	0	0
E15	1	1	1	1	1	0	0	0	1	1	1	1	1	1	1	1
E16	0	0	0	0	0	0	0	0	0	0	0	0	0	0	0	1

After completing the construction of the reachable matrix, the influencing factors are hierarchically divided and decomposed based on the matrix. For the former, it refers to dividing each element into separate subsystems; As for the latter, it refers to decomposing the factors contained in the system into different levels. By using hierarchical decomposition, all influencing factors will be included in the same system. Prior to this, it is necessary to accurately divide the leading set and reachable set. The leading set Q (Ai) refers to the set of elements corresponding to the row with the number 1 in the column corresponding to factor Ai in the reachable matrix M, expressed as Q (Ai) = {Sj S}; The reachable set R (Ai) refers to the set of elements corresponding to the column with the number 1 in the row corresponding to the factor Ai in the reachable matrix M. It is represented as R (Ai) = {Sj S}, and Table 9 details the hierarchical division results.

**Table 9:** Hierarchical classification of influencing factors.

	Reachable set R	Pre set Q	Intersection A = R ∩ Q
Ecological management (E1)	1	1,2,3,4,5,6,8,10,11,13,15	1
Consumer behavior (E2)	1,2,3,4,5,9,10,11,12,13,14,15,16	2,4,5,8,10,13,15	2,4,5,10,13,15
Persuasive behavior (E3)	1,3	2,3,4,5,8,10,13,15	3
Citizen behavior (E4)	1,2,3,4,5,9,10,11,12,13,14,15,16	2,4,5,8,10,13,15	2,4,5,10,13,15
Individual environmental cognition (E5)	1,2,3,4,5,9,10,11,12,13,14,15,16	2,4,5,8,10,13,15	2,4,5,10,13,15
Environmental emotions (E6)	1,6,16	6	6
Verbal commitment (E7)	7	7	7
Actual commitment (E8)	1,2,3,4,5,8,9,10,11,12,13,14,15,16	8	8
Natural environment knowledge (E9)	9	2,4,5,8,9,10,13,15	9
Environmental knowledge (E10)	1,2,3,4,5,9,10,11,12,13,14,15,16	2,4,5,8,10,13,15	2,4,5,10,13,15
Environmental action knowledge (E11)	1,11	2,4,5,8,10,11,13,15	11
Difficulty of behavior (E12)	12	2,4,5,8,10,12,13,15	12
Social regulations (E13)	1,2,3,4,5,9,10,11,12,13,14,15,16	2,4,5,8,10,13,15	2,4,5,10,13,15
Economic conditions (E14)	14	2,4,5,8,10,13,14,15	14
Environmental responsibility (E15)	1,2,3,4,5,9,10,11,12,13,14,15,16	2,4,5,8,10,13,15	2,4,5,10,13,15
Sense of environmental control (E16)	16	2,4,5,6,8,10,13,15,16	16

**Figure 3:**
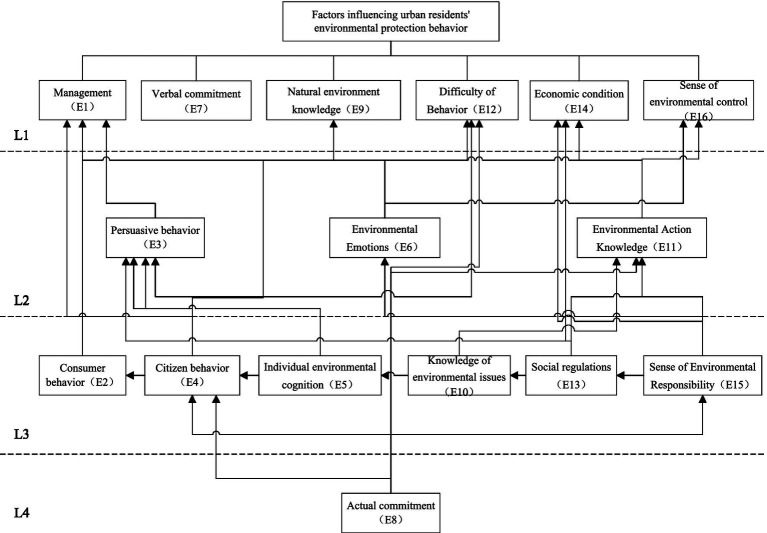
Structural model of factors influencing behavior explanation.

Assuming the intersection is C (Ai), when R (Ai) Q (Ai) = R (Ai), that is, when C (Ai) = R (Ai), the factors that meet this condition belong to the first layer of factors. After removing this layer of factors and repeatedly screening, each influencing factor is decomposed into different levels (Figure 3). Table 10 details the decomposition results.

**Table 10:** Hierarchical decomposition of influencing factors.

level	element
1st layer (top layer)	Ecological management, verbal commitments, knowledge of the natural environment, difficulty of behavior, economic conditions, sense of environmental control
Layer 2	Persuasive behavior, environmental emotions, environmental action knowledge
Layer 3	Consumer behavior, civic behavior, individual environmental cognition, knowledge of environmental issues, social regulations, and sense of environmental responsibility
4th layer (bottom layer)	Actual commitment

The explanatory structure model of factors affecting urban residents’ environmental protection behavior includes four levels, from high to low, namely L1, L2, L3, and L4. Among them, the L4 level is at the bottom level, and the influencing factors contained in this level belong to the fundamental factors; The L2 and L3 layers are located in the middle layer, and the influencing factors contained in this layer belong to indirect factors; The L1 layer is at the highest level, and the influencing factors contained in this layer belong to direct factors.

1 Analysis of direct influencing factors

The direct factors are at the highest level, also known as surface factors, and the identification difficulty of these factors is relatively small. Whether they improve has a direct impact on the environmental protection behavior of urban residents. The improvement of surface factors can enhance the environmental protection efficiency of residents in a relatively short period of time (Zhao, 2025). There are a total of six surface factors in the model, namely ecological management (E1), verbal commitment (E7), knowledge of the natural environment (E9), difficulty of behavior (E12), economic conditions (E14), and sense of environmental control (E16). Among them, verbal commitment (E7) and knowledge of the natural environment (E9) are independent factors, and other factors are difficult to influence them, so independent analysis is needed. Ecological management (E1), behavioral difficulty (E12), economic conditions (E14), and environmental control perception (E16) all affect environmental protection and behavior. To improve environmental protection effectiveness, it is necessary to start from these factors. Natural environmental knowledge (E9), economic conditions (E14), and behavioral difficulty (E12) are outcome factors that are easily influenced by other factors and also belong to surface level factors, and generally result factors are at the surface level.

2 Analysis of indirect influencing factors

Indirect factors are located in the middle layer, serving as a bridge connecting the highest and lowest layers. The fundamental factors at the lowest layer utilize the indirect factors in the middle layer to influence the surface factors at the highest layer. The intermediate indirect factors include persuasive behavior (E3), environmental emotion (E6), environmental action knowledge (E11), consumer behavior (E2), civic behavior (E4), individual environmental cognition (E5), environmental problem knowledge (E10), social regulations (E13), and environmental responsibility (E15). Among them, persuasive behavior (E3), environmental emotions (E6), environmental action knowledge (E11), civic behavior (E4), individual environmental cognition (E5), environmental problem knowledge (E10), social regulations (E13), and environmental responsibility (E15) all directly affect ecological management (E1), indicating that urban residents’ environmental protection cognition, emotions, social norm perception, and specific behaviors themselves (such as persuasion and civic behavior) are important and direct driving forces that cannot be bypassed in the implementation of ecological management, reflecting their core hub role in environmental protection impact behavior (Han and Zhang, 2025). Environmental emotions (E6), environmental action knowledge (E11), consumption behavior (E2), civic behavior (E4), individual environmental cognition (E5), environmental problem knowledge (E10), and social regulations (E13) all directly affect the sense of environmental control (E16), indicating that urban residents’ environmental cognition, emotional identification, knowledge mastery (action and problem), practical behavior (consumption and citizenship), and perceived social norms jointly shape their beliefs and confidence in influencing environmental effectiveness, highlighting their key position as a bridge between cognition and behavior.

3 Fundamental influencing factor analysis

The fourth layer belongs to the fundamental factor, which has a long-term and lasting impact on other factors in the system. It is the source that affects the environmental protection behavior of urban residents. If this factor is not taken seriously, it is difficult to improve the environmental protection effect from the root. The underlying factor is actual commitment (E8), which indicates that the root of environmental protection behavior lies in the inherent identification and responsibility solidification of residents towards the environment. Actual commitment (E8), as a fundamental factor, is essentially a manifestation of individuals internalizing environmental protection as their own values and meaning in life. It goes beyond short-term stimuli (such as knowledge dissemination or regulatory constraints) and drives individuals to actively form deep behavioral motivations (Deng, 2025). If this value recognition is lacking, the emotional guidance (E6), knowledge transmission (E10/E11), and even regulatory enforcement (E13) at the middle level will be like rootless trees, difficult to transform into lasting civic action (E4) or a true sense of environmental control (E16), ultimately leading to the superficial effectiveness of ecological management (E1). Grasping the ‘practical commitments’ is the foundation for activating the long-term mechanism of residents’ environmental protection behavior.

### 3.4 Analysis of MICMAC model results

**Figure 4:**
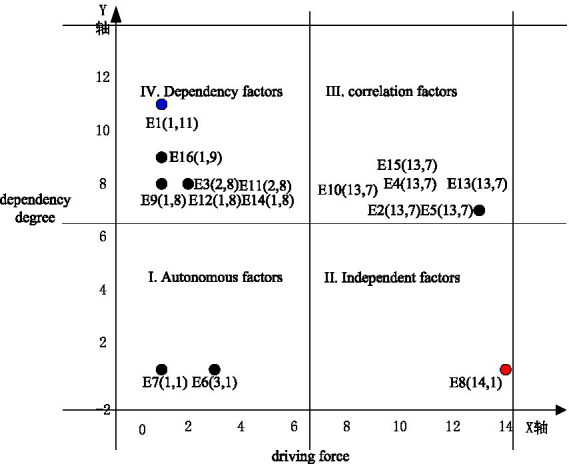
Quadrant diagram of driving torque dependence of influencing factors.

Using the MICMAC model, a reachable matrix M is established, and the values of each row and column in the matrix are summed up to obtain the values of driving force and dependence. Driving force refers to the degree to which the factor affects other factors, while dependence refers to the degree to which the factor is affected by other factors. Draw a quadrant chart using Excel tools, with the horizontal and vertical axes representing driving force and dependence, respectively, and set the mean of the driving force and dependence chart as the boundary. The quadrant chart divides the influencing factors into four categories: autonomous factors, independent factors, related factors, and dependent factors, which are located in quadrants I, II, III, and IV of the quadrant chart, respectively. Table 11 provides a detailed list of the driving forces and dependence values of each influencing factor, while Figure 4 intuitively shows the specific positions of each influencing factor in the quadrant chart.

1 Analysis of autonomous factors

**Table 11:** Driving forces and dependence values of influencing factors.

Influencing factors	Driving force	Dependency degree
Ecological management (E1)	1	11
Consumer behavior (E2)	13	7
Persuasive behavior (E3)	2	8
Citizen behavior (E4)	13	7
Individual environmental cognition (E5)	13	7
Environmental emotions (E6)	3	1
Verbal commitment (E7)	1	1
Actual commitment (E8)	14	1
Natural environment knowledge (E9)	1	8
Environmental knowledge (E10)	13	7
Environmental action Knowledge (E11)	2	8
Difficulty of behavior (E12)	1	8
Social regulations (E13)	13	7
Economic conditions (E14)	1	8
Environmental responsibility (E15)	13	7
Sense of environmental control (E16)	1	9

There are two factors in the first quadrant, including environmental emotions (E6) and verbal commitments (E7), which belong to autonomous factors. The driving force and dependence of autonomous factors are relatively small. Verbal commitment (E7) is a surface level factor, located in the L1 layer, and is not affected by other factors. It is relatively independent and has a weak impact on the system. The driving force and dependence of environmental emotions (E6) are significantly lower than the system mean, indicating that although it has weak behavioral guidance potential, it is almost unaffected by other factors and is in a relatively isolated “emotional island” state in the system. This low interactivity indicates that relying solely on emotional arousal is difficult to effectively transform behavior (E2/E4), let alone touch fundamental commitments (E8). The double low value of oral commitment further exposes that public statements can neither drive the chain of substantive behavior, nor are they changed by cognitive, knowledge or regulatory factors, forming a “commitment foam” that is separated from the core behavior mechanism. Environmental emotion (E6) is located in the middle layer of the model and has a significant impact on environmental protection behavior. This study suggests that autonomous factors are located at the highest level of the model, and these factors have relatively small influence and centrality. The analysis results of DEMATEL, ISM, and MICMAC are basically consistent, and should be identified as factors that have a weaker impact on residents’ environmental protection behavior.

2 Independent factor analysis

The second quadrant factors include actual commitments (E8), which are independent factors with strong driving force and weak dependence. They are key factors affecting the environmental protection behavior of urban residents, and their ultra-high driving force (14) and extremely low dependence (1) verify the fundamental role of the underlying factors in the ISM model. This ultra-high driving force means that actual commitment - that is, the real investment of individuals in environmental protection actions - has the strongest systematic radiation power, which can directly activate related factors such as consumer behavior (E2) and citizen behavior (E4), and even penetrate multiple layers of intermediaries to affect surface ecological management goals (E1); And its almost zero dependence (1) indicates its high degree of autonomy, unaffected by external factors such as environmental knowledge (E9-E11), social regulations (E13), or emotional commitments (E7). This stability makes it the most reliable behavioral leverage point in the system. However, this independence also hides governance paradoxes: on the one hand, policies can directly drive the overall situation efficiently by strengthening actual commitments, without relying on complex intermediary chains; On the other hand, if we overly focus on this factor and ignore its synergy with related factors, we may fall into the risk of “behavioral overdraft” - when the public is forced to commit without deep value internalization, it can easily lead to unsustainable behavior (Yang et al., 2025). Therefore, the independent nature of E8 is not only the fulcrum of system reform, but also warns that it needs to be cultivated with a sense of responsibility to form a “commitment responsibility” dual core drive, so as to avoid the high driving force becoming a short-lived behavioral foam.

3 Analysis of related factors

The third quadrant factors include consumer behavior (E2), civic behavior (E4), individual environmental cognition (E5), environmental problem knowledge (E10), social regulations (E13), and environmental responsibility (E15), which are related factors with strong dependence on driving torque. Changes in these factors can easily affect other factors, and changes in other factors can also lead to their impact. These factors are located in the middle layer and serve as a bridge connecting the bottom and top factors. Among them, the impact and centrality of environmental responsibility (E15) far exceed other factors, ranking first. The impact and centrality of civic behavior (E4) rank second, and the causality of individual environmental cognition (E5) ranks second. Therefore, it is necessary to focus on factors such as environmental responsibility, civic behavior, and individual environmental cognition to improve the environmental protection effect of urban residents.

4 Dependency factor analysis

The fourth quadrant factors include ecological management (E1), persuasive behavior (E3), natural environmental knowledge (E9), environmental action knowledge (E11), behavioral difficulty (E12), economic conditions (E14), and environmental control sense (E16). These factors belong to the dependent factors, located in the middle and upper levels of the model, with relatively small driving forces and high dependence. Other factors will have a significant impact on them. Among them, ecological management (E1), natural environmental knowledge (E9), behavioral difficulty (E12), economic conditions (E14), and environmental control sense (E16) belong to the top-level factors, while persuasive behavior (E3) and environmental action knowledge (E11) belong to the second level factors. Layer, consistent with the actual model. The causal degree values of these factors are relatively small, among which the causal degree value of environmental control sense (E16) is less than 0, ranking last, which also indicates that this factor is highly influenced by other factors.

### 3.5 Comprehensive analysis of key influencing factors

Based on the analysis results of the factors influencing urban residents’ environmental protection behavior in the previous chapters, it is possible to accurately determine the interrelationships between different influencing factors and establish a multi-level hierarchical structure explanatory model. This model can intuitively display the interrelationships between various environmental protection influencing behavior factors. Therefore, in the following content, this article conducts a deeper analysis of the calculation results of DEMATEL-ISM-MICMAC through the multi-level hierarchical explanatory structure model.

#### 3.5.1 Fundamental influencing factor analysis

The fundamental factors affecting the environmental protection behavior of urban residents mainly include actual commitments (E8). According to the DEMATEL model results, this factor is the causal factor, ranking high in its impact, indicating that it has a significant impact on other influencing factors within the system and plays a fundamental role in influencing environmental protection behavior. The MICMAC results further indicate that the driving force behind the fundamental factors of actual commitment (E8) is higher than the degree of dependence. Overall, according to the ISM model results, the most critical influencing factor is actual commitment (E8). This indicates that the root of environmental protection behavior lies in the inherent identification and responsibility solidification of residents towards the environment. The essence of actual commitment is for individuals to internalize environmental protection as a reflection of their own values and life meaning. It goes beyond short-term stimuli and drives individuals to actively form deep behavioral motivations (Wu, 2024). If this value recognition is lacking, the emotional guidance (E6), knowledge transmission (E10/E11), and even regulatory enforcement (E13) at the middle level will be like rootless trees, difficult to transform into lasting civic action (E4) or a true sense of environmental control (E16), ultimately leading to the superficial effectiveness of ecological management (E1). Grasping the ‘practical commitments’ is the foundation for activating the long-term mechanism of residents’ environmental protection behavior.

#### 3.5.2 Analysis of indirect influencing factors

Among the factors influencing urban residents’ environmental protection behavior, the indirect influencing factors mainly include persuasive behavior (E3), environmental emotions (E6), environmental action knowledge (E11), consumption behavior (E2), civic behavior (E4), individual environmental cognition (E5), environmental problem knowledge (E10), social regulations (E13), and environmental responsibility (E15), totaling 9 influencing factors. According to the DEMATEL model results, the transition factor is mainly the outcome factor. Among them, the driving force and dependence of consumer behavior (E2), civic behavior (E4), individual environmental cognition (E5), environmental problem knowledge (E10), social regulations (E13), and environmental responsibility (E15) are all at a high level, indicating that these influencing factors can not only affect other factors, but also depend on the state and changes of other factors. The MICMAC results further indicate that transitional factors are generally associated factors, which is consistent with the results of the DEMATEL model. According to the ISM model results, persuasive behavior (E3), environmental action knowledge (E11), social regulations (E13), and environmental responsibility (E15) are the core intermediate nodes. Plays a role of underlying fundamental factors and influencing surface factors.

#### 3.5.3 Analysis of direct influencing factors

Among the factors influencing urban residents’ environmental protection behavior, the surface factors mainly include ecological management (E1), verbal commitment (E7), knowledge of the natural environment (E9), difficulty of behavior (E12), economic conditions (E14), and sense of environmental control (E16), totaling six influencing factors. According to the DEMATEL model results, the dependence of surface factors is higher than their driving force, strongly dependent on the state or changes of other factors. Among them, verbal commitment (E7) and knowledge of the natural environment (E9) do not affect other factors, nor are they influenced by other factors, and are mainly independent factors in the system. The MICMAC results further showed that the surface factors were mainly independent factors and dependent factors. The surface factors that belong to the dependent factors indicate that they belong to endogenous influencing factors and are influenced by other influencing factors. The surface factors that belong to independent factors indicate that they are exogenous factors and are not affected by other influencing factors, reflecting more characteristics of randomness and suddenness. Schwartz et al. (2020) revealed through in-depth interviews that the passivity of ecological management behavior often stems from insufficient internalization of community norms rather than simple individual choices, which explains its high dependence characteristics (Schwartz et al., 2020). Hurst and Stern’s (2020) focus group study suggests that the vulnerability of environmental control perception is not so much due to a lack of technological capabilities as it is due to a weakened sense of efficacy caused by a lack of institutional support (Hurst and Stern, 2020). These qualitative findings support the quantitative conclusion of this study that the essence of surface factors is the product of the interaction between structure and individuals. Mohammad et al.’s (2022) participatory observation further found that the so-called “randomness” independent factors are actually driven by situational social expectations, and their suddenness precisely reflects the uncured environmental values (Morad et al., 2023). These multiple methods collectively validate the core viewpoint of this study, that ecological management (E1) and environmental control perception (E16) appear the most frequently in transmission pathways and have a higher level of importance.

## 4 Conclusion, discussion, and prospects

### 4.1 Research conclusion

This study analyzed the influencing factors of urban residents’ environmental protection behavior through the DEMATEL-ISM-MICMAC combination model, and obtained the following research conclusions:

1. The analysis results of DEMATEL method indicate that the environmental responsibility (E15) and citizen behavior (E4) with the highest centrality are the hub nodes of the system, indicating that individual responsibility awareness and public participation behavior have a global driving effect on environmental action. This is highly consistent with the theory of value belief norms in the category of environmental awareness, which emphasizes that individual environmental values drive behavior by activating moral norms. This study confirms the core position of E15 environmental responsibility as a norm perception, and its 0.973 causality degree confirms the transmission mechanism of “values responsibility perception behavior.” At the same time, the high centrality of E4 civic behavior reflects the systematic influence of public domain behavior, indicating that environmental behavior is not only an individual choice, but also the result of internalizing social norms, providing empirical support for the theory of value belief norms.
2. The results of the ISM method analysis indicate that surface direct factors such as ecological management (E1) and environmental control perception (E16) are directly associated with behavioral effects, but highly dependent on mid-level support; Indirect factors include 9 factors such as environmental emotions (E6) and civic behavior (E4), forming a “cognitive emotional behavioral” transformation bridge. Among them, environmental responsibility (E15) and social regulations (E13) drive the surface layer by influencing behavioral nodes such as E4 and E10; the fundamental factor is only actual commitment (E8), which serves as a deep value anchor, directly dominating the mid-level cluster and indirectly controlling the entire system. This forms a deep dialogue with the planned behavior theory in the transmission mechanism environmental behavior theory, which holds that behavioral attitudes, subjective norms, and perceived behavioral control collectively determine behavioral intentions. This study not only verifies the key role of E16 environmental control perception in corresponding “perceived behavioral control,” but also reveals the value internalization pre driving mechanism that has not been fully explained in the theory of planned behavior through the transmission path from E8 actual commitment to E15 sense of responsibility, indicating that behavioral control needs to be based on value recognition and promoting the deepening development of this theory.
3. The analysis results of MICMAC method show that the actual commitment of independent factors (E8) is located in the second quadrant with ultra-high driving force and the lowest dependence, becoming the only autonomous engine in the system; The cluster of related factors includes six factors, namely consumer behavior (E2) and environmental responsibility (E15), which combine high driving force and moderate dependence, forming a core lever group for behavior transformation; The dependence on factors such as ecological management (E1) and environmental control perception (E16) far exceeds the driving force, revealing the passivity of achieving surface level goals; The dual low values of autonomous factor environmental emotion (E6) and verbal commitment (E7) expose its systemic marginalization - emotional arousal and symbolic commitment are difficult to link with the main behavioral chain. This is in line with the innovative activation model of norms, which emphasizes the behavioral driving role of individual norms after the activation of perceived consequences and attribution of responsibility. This study reveals the systematic differences between genuine and verbal commitments through a sharp contrast between E8 and E7. At the same time, the “island effect” of E6 environmental emotions indicates that pure emotional arousal is difficult to activate the normative chain and must rely on the synergistic effect of E15 sense of responsibility. This provides important boundary conditions for the applicability of normative activation models in complex systems.
4. Through the DEMATEL-ISM-MICMAC combination model analysis, it is shown that the actual commitment (E8), as the fundamental driving source, is primarily determined by the ultra-high driving force and extremely low dependence characteristics displayed by MICMAC analysis. This makes E8 the only independent factor in the system, with the ability to autonomously influence the global situation. The ISM model further confirms its deep anchor position, indicating that E8 is located at the foundation of the hierarchical structure of influencing factors, serving as the core of value internalization and supporting the entire behavioral transmission system. The DEMATEL analysis results strengthen this judgment from a causal perspective, and the strong causality of E8 indicates that it has a significant radiating influence on other factors. The pivotal position of the cluster of related factors is reflected in high centrality (DEMATEL), intermediate transmission position (ISM), and dual high interactivity (MICMAC), which need to be activated through the “regulatory strengthening responsibility cultivation citizen action” triangle framework; The vulnerability of surface target ecological management (E1) stems from the triple identification of ultra-high dependence (MICMAC), outcome factor attribute (DEMATEL), and top-level position (ISM), which suggests that policies need to avoid simple outcome indicator assessments and instead strengthen mid-level capacity building. The conflict point of the model lies in environmental emotions (E6): it serves as a key middle layer in ISM, but MICMAC shows its isolation, implying that emotional intervention needs to be linked to E15 sense of responsibility design in order to break through the “island effect.”

### 4.2 Research discussion

Based on the above research conclusions, in order to ensure the effectiveness of environmental protection for urban residents, further research on countermeasures can be conducted from the following three perspectives.

#### 4.2.1 Deepening the internalization project of environmental protection value

As the fundamental driving factor, the independence and strong driving force of actual commitment (E8) require policies to focus on the internalization and cultivation of behavioral values. Establish a multi-level mechanism for converting environmental commitments and strengthen behavior internalization through community practice. Establish an “environmental practice points” system at the community level, where residents can participate in garbage classification supervision, low-carbon travel advocacy, and other practices to obtain priority for public services; Implementing “green seniority” certification at the enterprise level, employees can accumulate career development points by continuously practicing energy-saving and emission reduction behaviors; The education system incorporates environmental protection practices into comprehensive quality records, and students are required to complete annual ecological service hours (Lu, 2024). Synchronize the implementation of the “Commitment Action” tracking plan, with the street office regularly publicizing household environmental behavior data and matching personalized feedback reports, using community bulletin boards and digital platforms to display typical cases. Implement a ritualistic action for environmental protection commitments, embed a collective environmental protection oath segment in the occupancy ceremony of newly built communities, and issue star certification marks to families who fulfill their commitments. The government needs to establish a special support fund to provide green infrastructure upgrade rewards to communities with high commitment conversion rates, forming a closed loop of “value recognition behavior solidification continuous incentives.”

#### 4.2.2 Building a responsibility sharing action network

Build a collaborative framework of regulatory empowerment, responsibility concretization, and knowledge contextualization to address the high interactive characteristics of mid-level related factors such as consumer behavior (E2) and environmental responsibility (E15). Build a “chain of responsibility” governance system to activate the synergistic effect of social norms and environmental responsibility. Legislating to clarify the environmental responsibility transmission mechanism of enterprises in the circulation of goods, requiring e-commerce platforms to label product carbon footprints and associate them with consumer emission reduction contribution values. Establish an “Environmental Protection Conference Hall” system in the community, where resident representatives, property management, and merchants jointly formulate responsibility agreements, establish a garbage classification responsibility grid, and publish a red and black list of compliance. Implement the “Responsibility Visualization” project, develop a community level environmental responsibility map mini program, display in real-time the contribution value of residents’ water-saving, energy-saving, low-carbon travel and other behaviors, generate annual responsibility reports and include them in the credit incentive system. The ESG rating of enterprises should include supply chain responsibility transmission indicators and incorporate the environmental behavior of downstream distributors into the assessment. The government establishes a cross departmental responsibility coordination center, integrates environmental protection, education, and municipal supervision data to construct a resident responsibility portrait, and targets high responsibility perception groups^3^. Open up green credit discounts^4^. And the public service fast track.

#### 4.2.3 Optimizing behavior support infrastructure

High dependency factors such as ecological management (E1) and environmental control perception (E16) require systematic facilities and institutional support. A lightweight behavioral support system should be constructed for surface dependency factors to reduce the threshold for ecological management behavior. Set up “environmental service stations” in urban communities, providing shared tool rental (such as garbage classification smart bins, old object renovation equipment), one-stop recycling and disposal, and behavior guidance services. The operation of the stations is undertaken by third-party social organizations through bidding. Implement the “Green Consumption Voucher” plan, provide tiered subsidies for residents who purchase energy-saving certified products based on carbon emission reductions, and implement automatic deduction through joint payment platforms. Establish an environmental behavior adaptability certification system, simplify the compliance approval process for household photovoltaic installation, rainwater recycling, etc., and provide technical support services by the street office. Develop a “Behavior Convenience Index” monitoring platform to display real-time data on the coverage rate of garbage classification facilities and the efficiency of bus connections in each community. Prioritize the deployment of mobile recycling vehicles and shared bicycles for low zone areas. The government needs to include behavior support facilities in the mandatory standards for new community planning and establish a special bond fund pool for the renovation of old communities.

### 4.3 Future prospects

This study constructs a three in one framework of “driving mechanism transmission path governance strategy” using the DEMATEL-ISM-MICMAC combination model, which demonstrates significant differences from traditional research in future urban environmental governance. In the past, DEMATEL-ISM-MICMAC applications in the field of environment or city often focused on single dimensional analysis, such as isolated examination of social structure or psychological factors, resulting in fragmented conclusions and a lack of systematic linkage; And this study achieved the organic integration of multi-level factors, revealing the complete transmission chain from fundamental commitment to surface behavior. Future research can further expand the cross domain applicability of this framework, such as conducting longitudinal studies, studying the environmental protection behaviors of residents in different cities/countries, integrating real-time behavioral data with digital twin technology, dynamically simulating policy intervention effects, and surpassing traditional static analysis to provide more accurate and actionable decision support for global urban carbon neutrality, promoting the transformation of environmental governance from theoretical exploration to practical empowerment.

## Data Availability

The original contributions presented in the study are included in the article/supplementary material, further inquiries can be directed to the corresponding author/s.

## Footnotes

^1^It is to reduce the impact of personal activities on climate change by choosing transportation modes with low carbon emissions. Specifically, it includes the use of walking, cycling, or public transportation, bike-sharing and other means of travel. For necessary medium and long-distance travel, it advocates the use of new energy vehicles or carpooling to improve transportation efficiency.

^2^A special kind of artificial ecosystem is formed by the interaction between urban residents and their surrounding biological and abiotic environments. It is not formed naturally, but is established on the basis of human transformation and adaptation to the natural environment, and its core feature is that human activities are the leading factor.

^3^Evaluate the high-responsibility-aware groups through indicators such as environmental protection, consumption, educational participation and compliance and trustworthiness. Environmental behavior tracks the accuracy of household waste classification, household energy consumption data, low-carbon travel frequency and other indicators through the smart community platform. Consumer behavior integrates the data of the municipal supervision, scans the residents’ performance in the proportion of green products purchased and reduces the use of disposable products. In addition, the frequency of residents’ participation in community environmental education activities, their contribution to public consultation on environmental issues, and whether their daily behaviors are consistent with environmental commitments should all be included in the investigation of integrity and compliance.

^4^Green credit preference is a set of combined incentive tools. For the loan application for purchasing first-class energy-efficient household appliances, new energy vehicles or residential energy-saving renovation services certified by the state, interest rate discounts can be provided, which can be reduced by 50–100 basis points on the basis of the benchmark interest rate of the central bank, and the evaluation fees and handling fees in the process of loan handling are exempted. In terms of quota support, a higher credit line can be granted, which is specially used for green consumption and family environmental protection investment. The repayment method is also more flexible, supporting monthly interest payment, repayment of principal when due or customized repayment plan according to family seasonal cash flow.
